# The impact of COVID-19 on children and adolescents’ mental health

**DOI:** 10.15537/smj.2022.43.11.20220481

**Published:** 2022-11

**Authors:** Tamara A. Hafiz, Ahmed H. Aljadani

**Affiliations:** *From the Department of Health Promotion and Health Education (Hafiz), Faculty of Public Health & Informatics, Umm Al-Qura University, Makkah; and form the Department of Psychiatry (Aljadani), Faculty of Medicine, University of Hail, Hail. Kingdom of Saudi Arabia.*

**Keywords:** COVID-19, children, adolescents, psychological impact, mental health, interventions

## Abstract

Children and adolescents are more susceptible to the formation of mental health issues, since their brains are still developing. The coronavirus disease 19 (COVID-19) pandemic response measures have disrupted daily life and left individuals socially isolated, including children and adolescents. In view of this, this study aimed to give a narrative review of the literature on the pandemic’s effects on children’s and adolescents’ mental health, associated risks, and successful intervention strategies. There are still issues to be resolved to give children and adolescents in many regions of the world with quality, rights-based, and culturally relevant mental health care. It is difficult to predict how the COVID-19 pandemic may affect children’s and adolescents’ mental health in the short- and long-term. To address the mental and social health needs of children and adolescents after the pandemic, it is urgently necessary to perform longitudinal and developmental studies and introduce evidence-centered action plans and interventions.

**T**he coronavirus disease 19 (COVID-19) outbreak could have seriously harmed the metal health of millions of children and adolescents. As their brains are not yet fully developed, children and adolescents are at greatest risk of harm from stressful situations and social isolation. These factors can result in irreversible abnormal development. The unprecedented changes which accompanied the pandemic impacted every facet of life. This posed particular challenges not just for children and adolescents, but even more so for those among this cohort who have suffered with pre-existing psychiatric issues such as attention deficit hyperactivity disorder (ADHD), along with anxiety, depression, mood, and behavioral disorders.^1,2^ This therefore makes this a subject where the need for research exists.

The evaluation of mental health all the way from early childhood through adulthood is highly important, due to the knock-on effects which mental health has on other areas of life. The Centers for Disease Control and Prevention (CDC) highlights that mental health influences our emotions, thoughts, and behaviors, thus impacting how people make decisions, communicate with others, and handle stressful situations. Mental illnesses like anxiety, bipolar disorder, depression, and schizophrenia can have major impacts on those who suffer from them. The problem may be acute or chronic, with the changed mental state leading to significant behavioral changes and also impacting on physical health. Depression in particular has been shown to reduce capacities for rational thinking, as well as enhance the risk of other health problems like diabetes. This relationship works in reverse, too, in that sufferers of chronic diseases are at increased risk of mental health issues. This highlights how crucial it is to work toward healthy levels of physical and mental health, especially in children and adolescents.^3^

Traumatic life episodes include social isolation and witnessing ill health and death in others as well as oneself. These were all situations that children and adolescents could have experienced because of the pandemic, that would have had an adverse effect on their mental health, resulting in conditions such as stress, anxiety, as well as depression disorders. Post-traumatic stress disorder in particular can result from the pandemic situation, as children and adolescents may both fear for their own safety and of their families. The changes imposed by pandemic restrictions, including working and schooling from home, clearly highlight the seriousness of the situation, and can result in higher stress levels and sleep problems, among other issues such as substance abuse. For children and adolescents, an inability to plan for the future, disrupted academic and personal relationships, along with physical inactivity, are major risks to mental health which have resulted from the pandemic situation.^2,4-6^

The pandemic thus represents a critical juncture in the development of a whole generation of children and adolescents. It is imperative that society strives to ensure they do not become the big losers of the pandemic, but instead seeks to ensure their rights are promoted and protected. In a Saudi Arabian context, if this is not achieved, then the country will not be able to fulfill the goals it set itself in its 2030 Agenda, a developmental plan which aims to modernize and improve the Kingdom. Nevertheless, the nature and extent of the harm the pandemic inflicted differs from one child/adolescent to another, dependent on various factors such as developmental age, cognitive level, and existing mental and physical health. Those with pre-existing physical and mental health problems, disabled, those who live in deprivation, are especially at risk.^7^

This study therefore seeks to improve the mental health among children and adolescents. There are review articles which consider the impact of the COVID-19 pandemic. However, there is an absence of studies which both consider the impact of the pandemic as well as outlining effective evidence-centered interventions to preserve and improve the mental health of children and adolescents.

The objectives of the review: i) To understand how COVID-19 impacted children and adolescent’s mental health. ii) To determine what mental health risk factors are connected to the pandemic. iii) To outline evidence-centered interventions and practices for mental health protection and improvement among the target population.

## Discussion

The research strategy is split into 3 stages. *The preparatory stage involved:* a) Literature review: to be updated throughout the study.

b) Ethical consideration: ethical approval was obtained from the Medical Research Ethics Committee at Hail University, Hail, Saudi Arabia.

*The implementation stage involved:* a) Review articles on the impact of COVID-19 on the mental health of children and adolescents: From September 2021 to May 2022, a search was carried out on MEDLINE’s electronic databases through PubMed, and Web of Science. Search terms used included: “the impact of COVID-19 AND children,” “children AND COVID-19,” “psychological effects on children AND adolescents,” and “children mental health AND COVID-19.” To achieve a comprehensive and efficient review, modified terms were then also employed, including: “the impact of COVID-19 on children’s mental health” and “the impact of COVID-19 on the mental health of older children.”

b) Review articles on other aspects: Other search terms were used to identify related research, including: “violence against children AND COVID-19,” “children’s health AND COVID-19,” “chronic diseases children’s mental health,” and “evidence-based interventions for mental health recovery.” A manual search was also used to help find related studies and references. Case studies, guidelines, and reviews from the World Health Organization, United States (US) Department of Health, United Kingdom National Health Service, and American Psychiatric Association were also utilized.

c) Data extraction: Eligibility criteria

Inclusion criteria:

- Articles published in English language peer-reviewed journals.
- Mental health measured through validated clinical assessments or parent/child/teacher self-reports.

Exclusion criteria:

- Research concerning children and adolescent mental health which falls outside the COVID-19 pandemic period.
- Research focused on persons over eighteen years old or suffering from mental or psychological illness.
- Unpublished or non-peer reviewed research

*The evaluation stage involved:*

a) Double checking and revising the discussion and conclusions.
b) Recommendations, writing-up and publication

### Literature search results

The literature search results were uploaded to Mendeley site to screen against selection criteria, as well as to remove duplicate results. Following the initial search: (n=2147) possible studies were obtained, (n=478) of duplicate studies have been deleted. After titles and abstracts screening were studies excluded (n=1281). Of the 388 studies were evaluated in order to be assessed for eligibility. The articles excluded: no full text available (n=168), duplicated (n=44) and, irrelevant (n=161). A total of 15 studies were included in review.

That mental disorders are a significant cause of disability in children and adolescents around the world is well established in research literature.^8^ Moreover, though problems can occur throughout childhood, adolescents appear especially vulnerable. Underlining how this is a particularly sensitive period for the development of mental health problems, approximately 53,000 cases of adolescent suicide were recorded in 20160.^9^ Research has also found that in global terms, approximately 15% of children and adolescents suffer from mental disorders, with almost half of these disorders apparent by age 14 if untreated. Nevertheless, few children with mental disorders receive timely support.^8^

Depression is among the primary sources of mental illness in children and adolescents.^7^ In addition to this, over a billion children are exposed to violence annually, whether as a direct victim or indirect witness.^10^ Such experiences can have extremely harmful immediate as well as long-term impacts on mental health. For this reason, the 2030 Agenda for Sustainable Development included commitments to eliminate violence in all its forms, as well as enhance mental health and well-being. Every child must be free from violence and have the best possible mental health, according to the United Nations Convention on the Rights of the Child. However, there are still barriers to realization, such as a lack of funding and the ability to offer high-quality, human rights-focused, and culturally acceptable mental health care globally.

The COVID-19 pandemic served to exacerbate such challenges. The data, for now at least, remains indicative rather than demonstrative. However, what exists strongly supports the contention that preventive measures like school closures, social distancing, along with the disruption to home and work patterns and stress this caused, resulted in increased risk of children and adolescents being exposed to violence in the home. Furthermore, online platforms represent another area where children and adolescents may become exposed to violence, with greater periods of time spent online during the pandemic. Direct consultations have revealed that children and adolescents have expressed feelings of insecurity, fear, tension, and uncertainty because of the pandemic.^11,12^

By August 2020, 143 states in the US had elected to shut educational facilities, with over a billion students impacted, along with their families. These closures can cause new, or aggravate existing, mental health issues. Students are unable to fully engage in their studies or interact with their peers. Play, exercise, school activities, and communication with classmates are factors that are recognized to help in childhood mental growth and development, were significantly impacted by the pandemic.^13^ Parental mental health was also impacted by the financial and social pressures of the pandemic. Indeed, one survey from the US found that approximately 40% of parents whose children were under 12 years old reported that they experienced severe distress or difficulties balancing work and childcare responsibilities during the pandemic. Moreover, over half of the surveyed parents said social isolation and financial issues impacted their ability to provide the best level of care for their children.^14,15^

Data from China, found that during the lockdown in February 2020, domestic violence rates more than tripled. Research from Texas in the US also found that instances of suspected child abuse had increased, while in another survey parents reported that the amount that they were yelling and even slapping their children had increased during the pandemic period.^15-17^

## Mental health risk factors

The pandemic exposed children and their families to direct and indirect triggers of stress and emotional distress. It is not only the fear of infection which produces anxiety. Rather, the disruption to life and work patterns, difficulties in knowing how to react to an unprecedented context, along with the inability to escape from it, all impact on children and adolescent mental health, not least because of underdeveloped coping strategies.^2,5,18^

### Increased screen time

Children and adolescents were exposed to elevated levels of media coverage regarding the pandemic. They had harmful impacts, including causing excitement and anxiety, as well as helping to spread misinformation and rumors. Research found that following the September 11 attacks, the time spent watching screens for both children and teens increased. This in turn was linked with increase in post-traumatic stress disorder instances as well as other mental health problems. Increased usage of social media made also leaves children and adolescents more vulnerable to scammers and blackmailers, as well as to harmful or inappropriate content, even more so if their online activities go unsupervised.^19,20^

### Parental stress

Isolation at home has negative effects. Many families face significant pressures, while also struggle to find adequate support. Coping strategies include taking a walk and getting some fresh air, visiting a friend, going shopping or to the cinema, or going on a holiday. The inability to do many of these things during the pandemic exacerbated psychological stress.^20^

### Lack of physical activity and obesity in children

Many families found it difficult to put food on the table during the pandemic, because they usually rely on subsidized programs which provide their child with a least one healthy meal a day while in school. On the other hand, with others it is not insufficient calories that is the issue, but too many. Eating too much food which is of poor nutritional value, coupled with insufficient physical activity, is the main cause of childhood obesity. The pandemic restrictions exacerbated these issues. Obese children are in turn at greater risk of mental health problems like anxiety, depression, poor self-confidence and self-esteem, suicide and self-harm, substance abuse, along with physical health issues, and poor educational and employment outcomes.^21-23^

### Low opportunity and low resource

The pandemic did not spread evenly. Some lost loved ones, while others lived in places that were barely impacted by the virus. Some families have members who work on the front line in sectors like medicine and were thus more exposed to the virus, while others found themselves working from home. Others lost their jobs altogether, thereby placing them in a difficult financial position which can lead to mental distress. Children and teenagers, often found the transition to online learning to be a difficult and stressful process, especially those who did not have access to the necessary technology or did not have sufficient literacy to use it effectively.^24^

### Pre-existing comorbidities

Higher COVID-19 mortality rates are strongly correlated with pre-existing comorbidities. These include chronic issues like diabetes, high blood pressure, and cardiovascular disease (CVD). Some of those who caught COVID-19 also suffered from cardiovascular complications, including acute coronary syndrome, cardiomyopathy, myocarditis, arrhythmia, and stroke. Heart disease is recognized to have a potentially harmful impact on mental health. Anxiety and depression are both disorders which often coexist with coronary artery disease (CAD) and other CVD.^25^ Therefore, there is an important need to better understand these linkages within the particular context of the pandemic situation, so that effective preventative measures and treatments can be devised.

## Younger children

### Common symptoms of psychological distress in young children during the COVID-19 pandemic

Parents may observe their young children becoming more anxious. They may also squabble more while engaged in play. Young children are highly susceptible to parental pressure. Their reactions may come in the form of tantrums, aggression, opposition or other bad behavior, along with reactionary behaviors like repeatedly asking for a bottle or the toilet, refusing to get dressed or eat, thumb sucking, along with becoming more clingy, and seeking extra attention. They may also experience trouble getting to sleep or disturbed patterns of sleep, wherein they wake repeatedly during the night or have nightmares. An inability to nap during the day is another potential indication of distress. Doubt can creep into parents’ minds. They may believe themselves inadequate parents who do not understand their child, and as a result cannot control them. This in turn can lead to feelings of sadness and depression, along with insomnia, and other problems. Moreover, a distressed parent can in turn impact their child negatively.^20^

### Specific interventions for younger children to mitigate psychological stressors:

- If the child demands extra attention at bed or nap time, parents should endeavor to increase how much time they spend with their child during the day; for instance, they can take extra 10- to 15-minute breaks, which may be spent playing or singing with their child.^20^
- Younger children require more hugs than older children do.^20^
- Young children need to be prompted to wash their hands properly and frequently during playtime.^23^
- Children pick up and imitate speech, so news media should not be on the television when younger children especially are present, because they may be disturbed by events that they lack the life experience to contextualize properly.^20^
- Where possible, talking around the COVID-19 pandemic in front of younger children should also be avoided.^20^
- Video chat is beneficial for children; during periods of social isolation in particular, can help them to build and revive relationships.^20^
- When watching television or playing video games with children, it is beneficial to engage with them in respect what is happening.^20^
- Establishing a family routine (or if one is already in place, amending it where needed).^20^
- Encourage children to undertake different activities over the day, such as reading, drawing, jumping, climbing and singing.^20^

## Older children/teenagers/adolescents

### Common symptoms of psychological distress in older children/teenagers during the COVID-19 pandemic

Social relationships are especially important for older children. During the pandemic, their social life was significantly impacted by the measures adopted, which resulted in school closures, remote learning, and social distancing. This disruption can lead to feelings of frustration, nervousness, detachment, boredom, nostalgia, along with less energy, motivation, curiosity, and enthusiasm. The inability to attend events and see friends is particularly challenging, as it represents an important component of the crucial stage of social and psychological development which takes place occurs between the ages 12-19, as highlighted by the psychologist Eric Erikson.^20,26^

### Specific interventions for older children to mitigate psychological stressors:

- Plan daily activities, like studying, phone calls with friends, cooking, family time, and recreation.^20^
- Allocate time for outdoor exercise.^20^
- If outdoor exercise is not possible, it is good to sit by an open window, breathing in fresh air and looking at the daylight, while focusing on being calm and reflective.^20^
- Social interactions are especially important at times of enforced social isolation; online courses and communication with friends can be a substitute for face-to-face contact.^20^
- Good sleep has a beneficial impact on mental health; it is important to have an appropriate and consistent sleep and wake-up time.^20^
- Use of computers and smart phones before bed can produce insomnia and difficulty sleeping, because the bright (especially blue) light impacts melatonin production, which is a hormone that facilitates sleep.^20^
- If a daytime nap is required, this should not exceed half an hour, as any longer can make it difficult to get to sleep at bed time.^20^
- Feelings of sadness and frustration which arise from social isolation should not be suppressed; rather, it is good to talk about them, as well as look for ways to create replacement remote social events that can help to alleviate mental distress.^20^

## Children with special needs

### Common symptoms of psychological distress in children with special needs during COVID-19 pandemic

One in 6 children from the 2-8 age cohort were found to suffer from developmental behavioral or emotional difficulties. These include autism, ADHD, cerebral palsy, learning difficulties, and delayed development, among others. Children with special needs are especially susceptible to pandemic-related negative psychological effects, because of changes to their daily routine, school closures, and the disruption of their regular therapy sessions.^27-29^ Combined, these factors make it very difficult for children with special needs to access reference materials and learning opportunities, along with interact with peers, aspects which facilitate the development of important behavioral and social skills.^28^ School closures and insufficient access to therapists also makes it hard for parents, because they do not have the requisite professional experience in dealing with special needs children alone. This can in turn negatively impact the mental health of the parents, which may materialize as irritability and conflict with their teenage children.^18,30-32^

Just as the disorders differ, so do the needs of each individual child. Children with autism, for instance, can find it difficult to adapt to the changed pandemic setting and levels of self-harm may consequently increase. Suspending speech therapy and occupational therapy sessions can also harm skill development, with remote learning particularly difficult for children with autism.^31^ Moreover, children with ADHD find it difficult to comprehend what is going on around them as well as to understand cues from health care providers and other caregivers. They therefore find it hard to adhere to precautions and isolation, as it is difficult for them to be restricted to one place and not touch things, despite the fact they may spread infection if they do not follow guidance. Hyperactivity and heart rate may increase. This then makes it harder for children with ADHD to concentrate and pursue useful activities.^30^

### Specific interventions for children with special needs to mitigate psychological stressors:

- Maintain contact with therapists and schools.^28^
- Promote activities at home.^28^
- Parents of children with special needs face greater difficulties in respect their children’s education and may encounter mental distress themselves because of this, which can in turn impact their children; therefore, more consideration needs to be paid to what challenges these parents face and how to overcome them.^33^

### Specific interventions of families:

- Establishing an appropriate routine, with set meal and bed times, is encouraged by mental health professionals to help children with special needs to feel safe and in control.^20^
- Increased parent-child interaction, to strengthen the relationship between them.^34^
- Listening to children, giving them a safe environment in which to express their feelings and ask questions; children need reassuring and flexible relationships with their family, even more so at difficult moments.^20^
- Simple strategies and language should be used, which are age appropriate. This is especially important with younger children, who need to be taught why social distancing is necessary and why they cannot see friends and relatives like before, in a way which is intelligible to them. The need to adhere to preventive measures can be elaborated through phrases like: i)Help wipe things like toys and doorknobs to keep them clean. ii) There is no need to worry on people wearing masks; sometimes they wear them because they are not feeling well, but will stop when they feel better. iii) People sometimes get ill, but if you or your brother/sister do, your mom and dad will look after you.
- Parents or caregivers need to pay attention to their own mental health; the more confident in themselves and less anxious and stressed they are, the better the care they will be able to provide.^20^

## Specific interventions of educational institutions

The pandemic caused significant disruption to education, with most schools and colleges having to turn to remote learning via the internet. Teachers regularly interact with their students, which makes them well placed to assess their mental health. Several procedures and methods are available, including:

- Taking account of different students cognitive levels, teachers can look to inform them about COVID-19 and preventative health measures.^35^
- Teachers can utilize inventive ways to break the monotony of the school day and the impact this can have on mental health by engaging students with fun interactive activities like puzzles and mini quizzes, which can still have an educational theme.^35^
- Discussion sessions or virtual workshops provide an opportunity to talk about well-being and its importance, along with teaching life skills and simple exercises such as muscle relaxation, deep breathing, reflection, and positive self-talk that can enable children and adolescents to better deal with stress and anxiety.^35^
- Teachers should highlight the benefits of prosocial behavior along with the importance to develop students’ characteristics like tolerance and empathy. This will enable them to better prepare their students for life’s challenges and teach them that social distance need not ascribe to emotional distance.^35^
- Teacher-parent relationships can be established and strengthened via telephone and online communication, so that student mental health can be discussed even if face-to-face meetings are not possible.^35^
- Teachers should be in continuous communication with those parents whose children show signs of mental health issues, with the student referred to a mental health specialist, too.^35^
- Materials regarding life skills and other beneficial practices aimed at an academic audience should be made intelligible and accessible to students who do not enjoy internet access.^35^
- The ‘RAPID’ model of the John Hopkins PFA tool should be activated.^36,37^ This involves 5 stages: i) R - Rapport and reflective listening, to be implemented throughout. ii) A - Assessing and evaluating the individual’s psychological needs. iii) P - Prioritizing the needs based on severity. iv) I - Intervention to mitigate factors causing distress. v) D - Disposition and distribution of intervention to stabilize the survivor.
- Schools should provide support and World Health Organization guidance for students during lectures, to help protect and improve their mental health.^37^
- A licensed counselor should be employed to help students manage stress resulting from the pandemic. Both group and individual sessions may be used to inform students on coping strategies. These services should be made available in a timely manner, however.^37^
- Additional staff like public health counselors and school doctors should be assigned to help with stress management and provide support to students, which can help stabilize existing mental health problems while also preventing new ones from developing.^38–40^
- Comprehensive School Mental Health Systems to be introduced to help students. It is a school association which provides a different health-related services to students, informing them about such things are how to promote better health and prevent disease, how to detect disease early, along with evidence-centered practices. The help of community mental health counselors and physical health care providers is needed for this intervention.^38-40^
- The presence of a permanent technical team is needed to address any IT problems which arise during the implementation of interventions.^37^
- School must maintain constant contact with parents, with communication and expectations clear for both parties.^37^
- Schools should provide additional emotional education.^41^

## Other interventions

Along with the interventions already outlined, it is also necessary to encourage children to engage in healthy behaviors rather than unhealthy ones, as this improves problem-solving skills.^38^ Research found yoga to be beneficial for mental health and well-being, as well as stimulate positive behaviors in children and adolescents.^42^ Yoga is a holistic practice aimed at enhancing health by connecting mind, body and breathe. Physical postures (asanas) and breathing techniques (pranayama) are used for relaxation and meditation, with many pediatricians finding yoga to be an effective intervention.^43-45^ Yoga practice results in more balanced cortisol activity in the neurological and endocrine systems. This in turn decreases stress. The body produces less stress hormones such as adrenaline, noradrenaline, and cortisol. This makes people feel more positive and sleep better. Moreover, studies have found yoga is used in unique ways with respect to children. It has been employed to better regulate emotions, improve self-esteem, along with physical well-being, flexibility, self-control, and bodily awareness.^46–49^

## Conclusion

Insufficient interest in the short- and long-term psychological impact of the COVID-19 pandemic on the mental health of children and adolescents could lead many to suffer in silence. It is essential to understand the extent of the issue to devise effective strategies and interventions with which to tackle it. Such measures can deal with fears, helping to develop positive psychological behaviors which can lead children and adolescents to overcome such an unprecedented situation and its associated stressors.
